# “I Always Worry about What Might Happen Ahead”: Implementing Safer Conception Services in the Current Environment of Reproductive Counseling for HIV-Affected Men and Women in Uganda

**DOI:** 10.1155/2016/4195762

**Published:** 2016-03-08

**Authors:** Lynn T. Matthews, Francis Bajunirwe, Jasmine Kastner, Naomi Sanyu, Cecilia Akatukwasa, Courtney Ng, Rachel Rifkin, Cecilia Milford, Lizzie Moore, Ira B. Wilson, David R. Bangsberg, Jennifer A. Smit, Angela Kaida

**Affiliations:** ^1^Massachusetts General Hospital, Center for Global Health and Division of Infectious Disease, Boston, MA 02114, USA; ^2^Mbarara University of Science and Technology, Mbarara, Uganda; ^3^McGill University Research Centre, Montreal, QC, Canada H3H 2R9; ^4^Harvard T.H. Chan School of Public Health, Boston, MA 02115, USA; ^5^Massachusetts General Hospital, Center for Global Health, Boston, MA 02114, USA; ^6^University of the Witwatersrand, Maternal, Adolescent, and Child Health (MatCH) Research, Durban, KwaZulu-Natal 3629, South Africa; ^7^Brown University School of Public Health, Providence, RI 02903, USA; ^8^Simon Fraser University, Burnaby, BC, Canada V5A 1S6

## Abstract

*Background.* We explored healthcare provider perspectives and practices regarding safer conception counseling for HIV-affected clients.* Methods.* We conducted semistructured interviews with 38 providers (medical and clinical officers, nurses, peer counselors, and village health workers) delivering care to HIV-infected clients across 5 healthcare centres in Mbarara District, Uganda. Interview transcripts were analyzed using content analysis.* Results.* Of 38 providers, 76% were women with median age 34 years (range 24–57). First, we discuss providers' reproductive counseling practices. Emergent themes include that providers (1) assess reproductive goals of HIV-infected female clients frequently, but infrequently for male clients; (2) offer counseling focused on “family planning” and maternal and child health; (3) empathize with the importance of having children for HIV-affected clients; and (4) describe opportunities to counsel HIV-serodiscordant couples. Second, we discuss provider-level challenges that impede safer conception counseling. Emergent themes included the following: (1) providers struggle to translate reproductive rights language into individualized risk reduction given concerns about maternal health and HIV transmission and (2) providers lack safer conception training and support needed to provide counseling.* Discussion.* Tailored guidelines and training are required for providers to implement safer conception counseling. Such support must respond to provider experiences with adverse HIV-related maternal and child outcomes and a national emphasis on pregnancy prevention.

## 1. Introduction

Many men and women living with HIV in Uganda desire children [1–5]. Studies suggest that an estimated 10% of stable couples in Uganda are HIV serodiscordant and that such partnerships may account for up to 42% of incident HIV infections [6–9]. For HIV serodiscordant couples, attempts to achieve desired pregnancy also introduce a risk of HIV transmission to the uninfected partner.

Safer conception strategies include limiting sex without condoms to peak fertility, antiretroviral therapy (ART) with viral suppression for the infected partner, preexposure antiretroviral prophylaxis (PrEP) for the uninfected partner, and (for female-positive couples) home manual insemination and medical male circumcision. These strategies create opportunities for HIV serodiscordant couples to achieve pregnancy while minimizing periconception HIV transmission risks [10–12]. Implementation of such strategies may reduce HIV incidence, especially in settings like Uganda with high fertility rates (6.2 children per woman) and endemic HIV prevalence (8%) [13–15].

Access to safer conception strategies requires discussing reproductive goals with healthcare providers. In a recent study, 31% of HIV-infected men and women in care in Uganda reported wanting to have children, yet nearly two-thirds had never discussed their fertility desires with a provider [5]. Globally, client conversations with HIV care providers about pregnancy plans are infrequent, as shown in studies from sub-Saharan Africa [16–20], South America [21–23], North America [24–26], and Europe [27].

We used qualitative methods to explore healthcare providers' perspectives and current practice regarding reproductive counseling for women and men living with HIV and seeking HIV-related care in southwestern Uganda. The purpose of this research is to inform interventions that support HIV-affected individuals and couples to realize their childbearing goals while minimizing sexual HIV transmission.

## 2. Methods

### 2.1. Setting

We conducted in-depth semistructured interviews with outpatient healthcare providers in southwestern Uganda between May and December 2013. The Ugandan public sector healthcare system includes a tiered structure of health facilities, based on coverage and services offered [28]. The first tier, Health Centre I, refers to satellite health facilities and the Village Health Teams (VHTs), comprised of lay, voluntary village health workers primarily responsible for health promotion activities. Health Centre IIs are outpatient clinics staffed by nurses. Health Centre IIIs offer outpatient services and laboratory testing. Health Centre IVs offer inpatient services and minor surgery procedures. Regional hospitals provide comprehensive healthcare services. Provision of HIV clinical care is available at HC IIIs, HC IVs, and regional hospitals [29].

### 2.2. Sampling and Recruitment

#### 2.2.1. Health Centres

We compiled a list of HC IIIs and HC IVs in Mbarara District and used purposive sampling to select two HC IIIs (one rural and one semiurban), two HC IVs (one rural and one semi-urban), and the HIV treatment clinic at a regional hospital as interview sites (5 sites). Directors at selected health centres gave permission for staff participation in the study.

#### 2.2.2. Healthcare Providers

The nurse in charge provided a complete list of providers at each of the sampled health centres including medical officers (doctors who practice at HC IVs and regional hospitals), clinical officers (clinicians who provide health services up to and including minor surgeries and work in HC IIIs and higher), nurses, trained counselors, and village health team workers [30]. All medical officers and clinical officers were approached, given the small number of these providers. A stratified random sample of nurses, counselors, and village health team workers was sampled from each site, based on staff size. Study eligibility criteria included providing healthcare services for HIV-infected clients for at least six months. If a selected provider was ineligible, refused to participate, or was unavailable, we randomly selected another provider (from the same site and same type of provider, where possible) from the list. A total of 44 providers were approached for participation, of whom six declined or were ineligible to participate (participation rate = 86%).

### 2.3. Data Collection

Semistructured, in-depth interviews were conducted between May and December 2013. The research focus was to explore healthcare providers' assessment of reproductive goals among all HIV-infected male and female clients (not limited to known serodiscordant couples). We also explored providers' knowledge of safer conception strategies, attitudes towards people living with HIV having children, and provision of safer conception advice. Interview guides were developed by the authors and informed by a related study in South Africa [20]. Interviews were conducted until saturation was achieved, or until no novel, relevant topics appeared to emerge from the interviews [31].

Interviews were conducted in English or the dominant local language by research assistants fluent in both languages. Interviews were conducted in private, were audio recorded, and lasted approximately one hour.

### 2.4. Data Preparation and Analysis

Audio recordings of the interviews were translated into English, as necessary, and transcribed. Each transcript was reviewed for quality by study team members and corrections and clarifications were made where indicated.

Content analysis was used to assess providers' assessment of the reproductive goals of HIV-infected men and women, barriers and promoters to these conversations, advice offered, and knowledge of different safer conception strategies. Themes related to providers' assessment of reproductive goals among PLWH were uncovered and explored. An iterative process of reviewing and analyzing themes was performed using techniques described by Miles and Huberman [31]. The transcript coding team first identified major themes through independent review of a subset of transcripts. Coding was then performed to structure data into categories. Themes were then reexamined, and major and minor themes within each content area were identified. In order to ensure validity of the emergent data, themes were discussed with additional study team members including the research assistants who conducted interviews. Three coders (LTM, CN, and RR) analyzed the data independently. Results from each phase of analyses were compared and discrepancies were discussed until a resolution was reached. To check reliability of coding in the final phase, nine transcripts were coded by two coders each and then reviewed to resolve discrepancies before proceeding with coding the remaining data. NVivo10 software (QSR International) was used to organize the analysis.

### 2.5. Ethics

Participants provided voluntary, written informed consent at study enrollment. Ethics approvals were obtained from the Institutional Review Committee, Mbarara University of Science and Technology (Mbarara, Uganda); the Partners Human Research Committee, Massachusetts General Hospital (Boston, USA); and the Research Ethics Board of Simon Fraser University (Burnaby, Canada). Study clearance was received from the Uganda National Counseling for Science and Technology and the Research Secretariat in the Office of the President.

## 3. Results

### 3.1. Demographic Characteristics

Interviews were conducted with 38 healthcare providers including 5 medical officers, 7 clinical officers, 14 nurses, 7 counselors, and 5 village health workers. 76% were women with median age of 34 (range 24–57) years and had completed their training a median of 6 years previously. Providers reported a range of 2–80 individual patient consultations per day, with a median patient visit of approximately 19 minutes (range 3 to 120 minutes) (Table 1).

### 3.2. Overview of Qualitative Findings

We describe the emergent themes under two primary categories. First, we discuss providers' current reproductive counseling practices for HIV-infected men and women. Emergent themes in this category (A) include that providers (A1) routinely assess the reproductive goals of HIV-infected female clients but not male clients, (A2) offer reproductive counseling focused on “family planning” for the prevention of pregnancy and perinatal transmission and maintenance of maternal health, (A3) empathize with the importance of having children for people living with HIV, and (A4) describe opportunities to counsel HIV serodiscordant couples. Second, we discuss provider-level challenges that limit implementation of safer conception counseling. Emergent themes in this category (B) include the following: (B1) providers struggle to translate reproductive rights language into individualized risk reduction messaging given their concerns about maternal health and perinatal and sexual HIV transmission risks and (B2) many providers are familiar with safer conception strategies but lack the comprehensive training and support required to counsel clients. 

*(A) Current Reproductive Counseling Practices*

*Theme A1: Providers Assess Reproductive Goals of HIV-Infected Female Clients But Not for HIV-Infected Men*. Providers routinely assess reproductive goals and desires of female HIV-infected clients. This nurse explains the following:

*I ask her, how many children do you want to have? And how often … or how long between the one child to another? … She can tell you I want to have … like if she has two, she can tell you I want to have four. And I have to … I want to have like every three years.* (Nurse, 42 years, Site B)

During consults, however, providers commonly assess client circumstances with respect to parity, socioeconomic status, and partnership dynamics and then offer reproductive counseling based on this assessment, rather than on the woman's expressed reproductive goals.

*I do it [assess reproductive goals] on family clinic days, I ask the mother … how many children they have, how many sexual partners … whether she is with a new partner or an old partner and strongly emphasize the need for them to have a quality life without having too many children to cut down on … costs, because being HIV positive they also have to take care of themselves. … and they need to reserve energy to look after those [children] who are already there.* (Medical officer, 30 years, female, Site A)

In contrast to the practice for female clients, providers rarely assess male client plans for having children.

* Not really unless they have those questions. If there is no question that is related, usually … it's usually hard to remember that … you ask a man [about plans for having children].* (Counselor, 34 years, male, Site C)

While providers acknowledge that men play a dominant cultural role in determining family size, they viewed men as playing passive roles in clinic-based family planning programs.

*[We do not talk to men] because, if you look at reports and everything, men […] are not active when it comes to family planning. So most of them seem to have this idea in their head that it's a woman's duty, the family planning. So you always think it's better to address a person whose duty it is, than someone who is passively involved.* (Medical officer, 27 years, female, Site A)

*Theme A2: Reproductive Counseling Focuses on “Family Planning” and Maternal and Child Health.* Providers' reproductive counseling for HIV-infected clients focuses on preventing unintended or mistimed pregnancies through promotion of contraceptive uptake. These messages are emphasized even if a female client reports a desire to have a child.

* I don't beat about the bush, because we need them to get on family planning and reduce unnecessary pregnancies. Usually I counsel them, “do you think you really, really, really need this baby? Can't you do without the baby? Can your husband come and we talk about it together?”* (Medical officer, 30 years, female, Site C)

*… there is a woman who wants to produce for the sake [of producing], whether she can manage children or not. … So that one, you explain to her the situation we are in nowadays, how she is supposed to have few children, plan them because now children who are not educated are not needed. She produces few, she manages them, educates, then she manages, then, tomorrow they become something. She should not produce many, and heap them there and she fails to educate them or dress them up. So she produces few children that she can plan for.* (Village health team worker, 33 years, female)

Providers emphasized additional reproductive counseling considerations including preserving maternal health (including maintaining maternal nutrition, high CD4 cell count, and suppressed viral load during pregnancy) and linkage to prevention of mother to child transmission services (PMTCT) to minimize perinatal transmission risk. However, there was little mention of preconception counseling aimed at reducing sexual transmission of HIV during pregnancy attempts.

*If they are HIV positive … there are so many factors affecting their … immunity, one is HIV. But even I think pregnancy sort of wears and tears on the body. So you have no space in between and yet usually they have to work for their transport to here, because they have these routine visits that they have to make, so I emphasize it a lot. That family planning is really beneficial to them. Now, in the era of PMTCT, it's not a question about you giving HIV to your child, it's a question of you being able to be productive, and having children who are spaced and really taking care of them.* (Medical officer, 27 years, female, Site A)

* I would talk about CD4. I would talk about their nutrition status … which is talked also in other clinics which are not HIV but for us our difference is that we talk about the CD4, and adherence to treatment. Because if you are going to get pregnant and you are HIV positive you have to take very well your medication, so as not to infect the unborn. And maybe I would talk about PMTCT even before they have these babies. They get pregnant they should know that there is a mechanism of preventing mother to child transmission.* (Nurse, 27 years, female, Site A)

*Theme A3: Providers Empathize with the Importance of Having Children for People Living with HIV*. Discussions about reproductive counseling for HIV-infected clients are facilitated by providers' empathy regarding the importance of childbearing, acknowledgment of the reproductive rights of PLWH, and expressions of “*a duty” *to support pregnancy decisions of HIV-affected couples, especially those without children.

*We would sit them [PLWH who wish to conceive] down and look at all the possible ways and then the decision is theirs. Because I would not tell someone that because you see you are positive, your wife is positive so you cannot have a baby. Because sometimes this is a young couple, they have not had any children so they have the right to have a child. So we look at [the] problem, the advantages, the disadvantages [and] the benefits, and then the decision is theirs.* (Nurse, 38 years, female, Site A)

Several providers, including this counselor, spoke of evolving attitudes towards childbearing among PLWH over time, including a shift from discouraging pregnancy to advising on the general clinical conditions under which to attempt pregnancy.

* So people would keep quiet up to the time they are giving birth and we don't know, because they were fearing our condemnation. But we talked about it as a team, like, “you people, we need to change our attitude.” Now we tell them it's ok to give birth but you're going to do it at this CD4, so now they know. They keep asking me “is my CD4 right - can I go for a pregnancy now?”* (Clinical officer, 32 years, female, Site C)

*Theme A4: Providers Describe Opportunities to Counsel HIV Serodiscordant Couples*. Providers from several clinics described opportunities for counseling men and women together during group counseling sessions for serodiscordant couples and during family clinic days as described below:

*Yeah, actually the women come, we have a [sero] discordant [couples] meeting … here … the women who are negative come in and tell you, “Mushaho [doctor], talk to him he forces me sex without a condom, you know.”* (Clinical officer, 32 years, female, Site C)

*And these mothers, who are positive, we also carry out what we call family support groups. We bring them [mothers, children, spouses] together … to share their experiences as clients … and it also helps us care for them right.* (Nurse, 42 years, female, Site B)

In summary, healthcare providers in Mbarara District regularly assess reproductive goals among HIV-affected women, but not men. In this counseling, providers emphasize the importance of limiting and spacing children, preserving maternal health, and limiting perinatal HIV transmission risk and demonstrate an evolving empathy with PLWH's desires to have children. In addition, through government and NGO programs, providers have opportunities to offer family-based and couples-based counseling. Thus, although specific counseling about pregnancy planning and safer conception counseling is rare, the background milieu of provider-initiated reproductive counseling for PLWH in Uganda appears conducive to safer conception programming.

*(B) Primary Challenges Impeding Providers from Implementing Safer Conception Strategies*

*Theme B1: Providers Are Concerned about Childbearing Risks for PLWH*. The language of “reproductive rights of PLWH” was used by nearly all providers, including the following nurse:

*We don't stop them from getting pregnant- from having such ideas, because it is their right. It is the right of women.* (Nurse, 32 years, female, Site A)

However, provider narratives reveal a tension between reproductive rights and personal attitudes towards childbearing among HIV-affected couples. Notably, provider experiences caring for PLWH influence the approach, which discourages pregnancy to avoid attendant risks of HIV transmission and poor maternal and child health outcomes.

*I would discourage her. [But] if it's her wish and it's her right to have children, I would tell her what she is supposed to [do] before she decides to become pregnant.* (Nurse, 42 years, female, Site B)

*As a person I feel they [HIV infected women] shouldn't have children … they shouldn't bear children, because we know … pregnancy further makes their immune system low, yeah it further compromises the immune system and therefore they, they deteriorate faster than a person who didn't really involve herself in things to do with pregnancy.* (Clinical officer, 34 years, male, Site A)

Providers also expressed discomfort raising the option of condomless sex to achieve pregnancy.

*We always tell them if you have this child [with a serodiscordant partner] and you have seroconverted it's not our problem. It's their, I mean, it's their choice to have a child. […] You tell them about the risks of having a child as a discordant couple: you are at risk of being seroconverted, you are at risk of having other STDs like syphilis and everything like gonorrhea. That means that you are not practicing any safer sex methods. You're just having live sex [condomless sex] which will bring in other risks.* (Nurse, 27 years, female, Site A)

This tension contributes to providers feeling compelled to express their support of reproductive rights and acknowledge that pregnancy among HIV-affected couples can be safely achieved, while maintaining reproductive counseling messaging that includes several caveats regarding the safety of pursuing pregnancy in the context of HIV.

* They [men and women living with HIV] should go ahead and have children but most importantly they should give birth in the health facility. … I always worry about what might happen ahead [when a woman with HIV says she would like to have a baby], that she may infect her husband with HIV. So I tell them at least you stop there and be happy with the children you have so that you do not find yourselves dying at a go. So I tell them to stop giving birth if it is possible. I tell them to stop there and be happy with the ones they have, not to find them to die at a go, so I advise them to stop giving birth if it is possible.* (Village health team worker, 32 years, female, Site E)

*Theme B2: Providers Are Familiar with Safer Conception Strategies But Lack Support to Provide Safer Conception Counseling*. We found that most providers understand the epidemiology of serodiscordance, have direct experience caring for HIV serodiscordant couples, and identify opportunities for HIV prevention within these couples, all of which are precursors to initiating conversations about achieving pregnancy while minimizing HIV transmission risks.

* Ok that is the discordancy we were talking about. Like there are chances of having HIV. It's not necessarily that the first time you have sex with someone, that that's [when] you will get the HIV. So you might find that people are sleeping together, the other one is HIV positive, and the other one is HIV negative. And mostly if my partner is on ARVs and his CD4 is really high, his chances of transmitting the HIV to me will be low. And if I don't have any sexually transmitted infection […] his chances still of transmitting the HIV to me will be low.* (Nurse, 24 years, female, Site B)

With the exception of village health workers, providers were also familiar with a range of specific safer conception strategies. Frequently discussed strategies included timing condomless sex to peak fertility, delaying sex without condoms until the infected partner is on ART with a suppressed viral load and/or high CD4 cell count, and treating STIs prior to conception attempts. Given provider experience with promoting ART adherence to suppress viral load in clinical care, providers expressed enthusiasm about ART as prevention with condomless sex timed to peak fertility to reduce sexual HIV transmission in the context of desired pregnancy.

*First of all … the HIV positive client has to be on ARVs, and then the viral load has to be as low as possible and then the CD4 has to be high, very high and they must have been using condoms. And then we, they, they should be free from these other sexually transmitted illnesses … so that when time comes, when that time comes we usually advise them to look at the fertility days … on the peak they can meet and then resume the use of condoms.* (Counselor, 37 years, female, Site C)

In addition, providers mentioned manual insemination for female-positive serodiscordant couples, linking its use with peak fertility. A few providers also recommended pre- or postexposure prophylaxis for the uninfected partner, explaining that “*those are the methods [PrEP] which have been very effective in helping discordant couples stay discordant” *(medical officer, 27 years, female, Site A). Some providers were familiar with laboratory-based assisted reproductive technologies such as sperm washing to support safer conception but acknowledged that these are largely inaccessible to most of their clients.

Despite technical knowledge about some safer conception methods, data revealed a gap in translating that knowledge into safer conception counseling. Providers noted a lack of formal training in reproductive health and the absence of guidelines as barriers to comprehensive reproductive counseling.

*That counseling [for serodiscordant couples wishing to conceive] is also hard, but we try. […] In most cases, you tell the negative man, of course to … you can't tell that person to leave the woman directly, but you ask for options, what do you think? […] If a woman is positive, they are not having children, of course. […] Sincerely, we always tell them to stay safe [use condoms], but if a man persists, you let them, you let him do it on his own risk.* (Nurse, 30 years, female, Site B)

*I: Have you received any training with regard to HIV and reproductive health counseling?*

*P: (Shakes head to mean no)*

*I: So how come you know all these things in HIV and reproduction, where did you get all the information? You are really equipped with knowledge.*

*P: It's not getting information, but it's like on-job training. … I think if we could all get training in reproductive health, we could do better.* (Nurse, 28 years, female, Site C)

In addition, the large number of clients seen per day and the necessarily short time spent in each encounter (Table 1) present an additional structural barrier for providers to deliver nuanced reproductive counseling.

Overall, clinic-based providers (including medical and clinical officers and most nurses and counselors) demonstrated understanding of HIV transmission risk and HIV prevention opportunities, awareness of safer conception strategies, and an interest in receiving additional training to support reproductive counseling for PLWH. In contrast, a limited understanding of HIV transmission risk among village health team workers remains a challenge to the delivery of community-based safer conception messaging.

## 4. Discussion

Through qualitative interviews with healthcare providers from five public sector clinic sites, we found that clinic-based providers in southwestern Uganda are primed to provide safer conception counseling to men and women living with HIV. While few providers offer such counseling, most routinely conduct reproductive counseling with female HIV-infected clients, understand HIV serodiscordance, are aware of strategies to reduce HIV transmission risk beyond condom use, and are empathetic to childbearing desires among PLWH. In addition, many clinics offer “family clinic days,” which provide support for HIV-affected families, including HIV serodiscordant couples. Despite these initiatives, a key barrier to the delivery of safer conception counseling is provider discomfort providing advice that reduces but does not eliminate risks of HIV transmission or poor maternal-child health outcomes. In Uganda, provider training and practice has emphasized the need to promote condoms for HIV prevention and family planning, both national public health priorities [32, 33]. Thus, despite recognizing the reproductive rights of PLWH and knowing technical details of safer conception strategies, healthcare providers in this setting are comfortable providing counseling on contraceptives and condom use but struggle to support condomless sex for achieving pregnancy among HIV-affected clients. However, our findings suggest that this barrier could be ameliorated through additional reproductive rights-based provider training on the use of strategies that reduce HIV transmission while allowing for pregnancy [10, 11].

These findings are consistent with those of a recent qualitative study examining knowledge about safer conception methods among providers in two urban Ugandan settings, which described provider enthusiasm to incorporate safe conception counseling into their practice, a high level of provider knowledge about condomless sex timed to peak fertility, and some knowledge about manual insemination. Similar to our findings, an emergent theme described provider discomfort with recommending uptake of non-condom-based strategies that do not eliminate risk of sexual HIV transmission [34, 35].

Our findings suggest fewer barriers to implementation of safer conception programming in Uganda compared with other settings. Studies from South Africa describe provider misunderstanding about HIV serodiscordance and low knowledge about safer conception strategies [20, 36]. Although not explored directly, given the high overall fertility rate in Uganda (TFR = 6.2) [15], some of this difference may be due to Ugandan providers' practice of regularly initiating conversations about pregnancy and family planning with clients, regardless of HIV status.

In Uganda, the importance of limiting and spacing childbearing is emphasized during client consultations. Although PLWH are largely dissuaded from having children, these routine discussions provide opportunity to extend conversations about family planning to include safer conception for HIV-affected clients who desire children. Previous studies have shown HIV-affected men and women are eager to have these conversations with providers but are not yet willing to initiate such conversations [34, 37]. Both providers and clients report that conversations about pregnancy in the context of HIV concentrate on contraceptive uptake to prevent pregnancy and/or PMTCT to prevent perinatal transmission. The integration of safer conception counseling to prevent sexual HIV transmission in the context of desired pregnancy is a key step towards the implementation of comprehensive family planning services for HIV-affected individuals and couples. As shown here, providers are motivated to help PLWH have healthy babies, and the timing to integrate safer conception messages is opportune given the scale-up of Option B+ in Uganda as well as recent WHO guidelines regarding initiating ART regardless of CD4 cell count [14, 38].

Additional efforts are required to better engage men in discussions about reproductive goals. Previous work has shown that HIV-infected male clients are eager to have these conversations with providers and that providers and policy makers are aware of the importance of engaging men in HIV prevention and family planning; however, providers have limited guidance and training regarding how to engage men [39, 40]. In Mbarara District, the existing family clinic days, couples counseling, and serodiscordant couples' groups provide important avenues for engaging men in HIV-related care and reproductive counseling.

Our description of high levels of knowledge and enthusiasm regarding safer conception strategies among some clinical providers, despite a lack of specific training, is encouraging. This finding again highlights the opportunity to support implementation of safer conception programming in Uganda. Such training must explicitly address the finding that offering safer conception counseling, which promotes condomless sex and pregnancy, runs counter to previous training and providers are conflicted about the harm reduction messaging at the core of safer conception counseling. Our data from a small sample of village health workers (*n* = 5) suggest that understanding of HIV transmission risks in the context of pregnancy was low. Thus, the development of a training program for community-based providers to deliver individualized reproductive counseling messages to PLWH is premature. Additional research on village health workers is required to understand the most appropriate training entry point to support community-based safer conception counseling.

Due to the fact that this was exploratory qualitative research, a small sample of providers was interviewed. As such, these findings may not fully reflect the knowledge and practice of general public sector healthcare providers regarding reproductive counseling and support for HIV-affected clients. In addition, 6 of the 44 approached providers chose not to participate; thus our sample may be biased towards a certain type of provider (perhaps more engaged, more interested in reproductive health, and/or less busy). Study strengths include enrollment of a range of providers working at three levels of healthcare facilities in both rural and urban settings.

### 4.1. Recommendations

Several recommendations emerge from these findings. The first is a need for national safer conception guidelines and comprehensive training for providers. South Africa is the only country in sub-Saharan Africa with safer conception guidelines, and although implementation of the guidelines within public sector clinics has been limited, demonstration projects reveal client demand for and uptake of provider-initiated safer conception services [12]. Data from demonstration projects will be critical to show effectiveness of safer conception counseling to reduce HIV transmission risks.

Training focused solely on the technical details of safer conception strategies will be insufficient. Rather, training must respond to provider experiences and concerns about adverse maternal and child outcomes and a national emphasis on pregnancy prevention and condom use. Training grounded in a community-informed and reproductive rights-based framework [41] may promote ongoing evolution in provider attitudes towards childbearing for PLWH and overcome the current gap between awareness and practice in meeting the reproductive care needs of HIV-affected clients.

Provider-initiated conversations about pregnancy plans among HIV-affected clients need to be more frequent and more open. Men and women living with HIV report feeling that providers are unsupportive of PLWH having children and are thus unlikely to initiate these conversations [4, 5, 16, 20, 35, 37]. Even if clients do not report plans to have children, routine reproductive health counseling should include preemptive messaging regarding the availability of strategies to achieve pregnancy while minimizing HIV transmission and risks to maternal and infant health. Consistent with current WHO treatment guidelines, all reproductive counseling should include messaging to wait to attempt pregnancy until the HIV-infected partner is on effective ART.

## 5. Conclusion

Safer conception services offered by healthcare providers can support people living with and affected by HIV to achieve desired pregnancy while minimizing risks to maternal, partner, and infant health. Our findings suggest that providers in Uganda recognize the reproductive rights of people living with HIV and have experience providing reproductive counseling to HIV-affected couples. The development of tailored safer conception guidelines and associated training for providers is the next step to translate emerging HIV prevention and treatment science into better health outcomes for HIV-affected couples desiring pregnancy. Such training must be responsive to provider experiences with adverse HIV-related outcomes associated with pregnancy and pregnancy attempts, cognizant of existing provider emphasis on pregnancy prevention, and acknowledge provider discomfort with counseling approaches that reduce but cannot eliminate HIV transmission risks. These findings underscore the need and opportunity to support providers to implement safer conception programming in Uganda.

## Figures and Tables

**Table 1:** Characteristics of participating healthcare providers (*n* = 38).

Characteristics	All (*n* = 38) *N* (%) Median (range)	Medical & clinical officers (*n* = 12) *N* (%) Median (range)	Nurses (*n* = 14) *N* (%) Median (range)	Counselors (*n* = 7) *N* (%) Median (range)	Village health workers (*n* = 5) *N* (%) Median (range)
Female	29 (76)	7 (58)	14 (100)	5 (71)	3 (60)

Age (years)	34 (24–57)	33 (26–51)	31 (24–38)	42 (34–57)	33 (24–45)

Years since completed training	6 (3–26)	10 (3–26)	5 (3–11)	6 (6–23)	5 (4–12)

Number of patients seen or community visits completed during last complete work day	30 (2–80)	53 (10–75)	30 (2–80)	12 (7–30)	6 (2–15)

Time spent in typical patient encounter or community visit (minutes)	19 (3–120)	11 (5–30)	18 (3–60)	23 (18–120)	60 (13–90)
